# Associative memory advantage in grapheme-color synesthetes compared to older, but not young adults

**DOI:** 10.3389/fpsyg.2014.00696

**Published:** 2014-07-14

**Authors:** Gaby Pfeifer, Nicolas Rothen, Jamie Ward, Dennis Chan, Natasha Sigala

**Affiliations:** ^1^Brighton and Sussex Medical SchoolBrighton, UK; ^2^School of Psychology, University of SussexBrighton, UK; ^3^Sackler Centre for Consciousness Science, University of SussexBrighton, UK; ^4^Department of Clinical Neurosciences, University of CambridgeCambridge, UK

**Keywords:** pair-associates, synesthesia, aging, visual perception, signal detection, learning, retrieval

## Abstract

People with grapheme-color synesthesia perceive enriched experiences of colors in response to graphemes (letters, digits). In this study, we examined whether these synesthetes show a generic associative memory advantage for stimuli that do not elicit a synesthetic color. We used a novel between group design (14 young synesthetes, 14 young, and 14 older adults) with a self-paced visual associative learning paradigm and subsequent retrieval (immediate and delayed). Non-synesthesia inducing, achromatic fractal pair-associates were manipulated in visual similarity (high and low) and corresponded to high and low memory load conditions. The main finding was a learning and retrieval advantage of synesthetes relative to older, but not to younger, adults. Furthermore, the significance testing was supported with effect size measures and power calculations. Differences between synesthetes and older adults were found during dissimilar pair (high memory load) learning and retrieval at immediate and delayed stages. Moreover, we found a medium size difference between synesthetes and young adults for similar pair (low memory load) learning. Differences between young and older adults were also observed during associative learning and retrieval, but were of medium effect size coupled with low power. The results show a subtle associative memory advantage in synesthetes for non-synesthesia inducing stimuli, which can be detected against older adults. They also indicate that perceptual mechanisms (enhanced in synesthesia, declining as part of the aging process) can translate into a generic associative memory advantage, and may contribute to associative deficits accompanying healthy aging.

## Introduction

Synesthesia is a stable perceptual phenomenon whereby one sensory stimulus (e.g., a visual word or auditory tone) leads to a secondary experience such as colors, tastes, smells, etc. Grapheme-color synesthesia in particular refers to the experience of seeing specific colors in response to particular letters, words, or digits (graphemes), e.g., “five is blue.” Recent studies have shown that people with grapheme-color synesthesia (hereafter referred to as synesthesia) have a memory advantage over control subjects matched for age, gender and education, especially for verbal stimuli that elicit a synesthetic color (Yaro and Ward, 2007; Rothen and Meier, 2010; Gross et al., 2011; Radvansky et al., 2011). The most prevalent and generic cognitive model to explain the synesthetes' verbal memory advantage (see Rothen et al., 2012 for a review) is the dual-coding theory (Paivio, 1991). According to this theory, more efficient and durable memory traces are obtained when words are additionally associated with visual images. Dual-coding effects can be observed in the normal population when using memory strategies such as associating words with locations in space [Method of Loci (Verhaeghen and Marcoen, 1996)] or using visual imagery, e.g., forming a mental picture of the words' meaning (Ishai and Sagi, 1997). Since synesthetes automatically activate visual images in the form of colors in response to words, this may serve as an explicit verbal memory aid and can explain the memory advantage for verbal material.

However, the dual-coding theory falls short of explaining empirical evidence of enhanced memory performance in synesthetes for visual stimuli that do not elicit a synesthetic color experience. Two types of stimuli, with and without color, have been tested in synesthetes. Regarding stimuli with color, Yaro and Ward (2007) were the first to show that synesthetes were significantly better than controls in memorizing colors arranged in matrices. Two additional studies, probing visual associative memory (VAM) with color stimuli further confirmed the selective color memory advantage in synesthetes relative to controls, which may not extend to other stimulus features, such as shape and location (Rothen and Meier, 2010; Pritchard et al., 2013). The memory advantage for color may stem from the synesthetes' frequent sensory experiences with colors following the secondary responses to words. These experiences in return sensitize color-processing areas in the brain and lead to enhanced color perception (Banissy et al., 2009). The reliable color memory advantage found in synesthetes therefore suggests that synesthetes might be “color experts” (Pritchard et al., 2013). Studies with stimuli that neither evoke a synesthetic response, nor contain a perceptual color, which would suggest a more general memory advantage in synesthetes, have reported mixed results. An advantage for synesthetes over controls has been reported with achromatic (black-and-white) abstract stimuli (Rothen and Meier, 2010; Gross et al., 2011; Ward et al., 2013), although others have not found this effect (Yaro and Ward, 2007; Pritchard et al., 2013). Likewise, figural recognition memory is enhanced in synesthetes (Rothen and Meier, 2010), while recognition memory for faces is not (Gross et al., 2011). Moreover, in assessing VAM, Gross et al. (2011) used achromatic abstract line-drawings paired with geometric shapes and found no significant retrieval difference between synesthetes and controls. One possibility for Gross et al.'s findings might have been an underpowered design, in which four synesthetes were tested, and all participants reached ceiling performance on the third trial, making it difficult to establish the potential memory advantages relative to controls. However, a second possibility is that the synesthetes' memory advantage for non-synesthesia-inducing stimuli is too subtle to be reliably detected against demographically matched control participants. It is worth noting that on average, the synesthetes outperformed the controls in all of the above reviewed studies, even though the differences were not always statistically significant.

How can the potentially subtle, generic memory advantages in synesthetes be explained? An alternative theory to dual coding and/or color expertise posits that the superior performance of synesthetes in declarative memory tasks stems from differences in their brain function or structure, e.g., increased white matter connectivity (Rouw and Scholte, 2007; Whitaker et al., 2014), or functional connectivity (Dovern et al., 2012). Functional brain differences between synesthetes and controls during perceptual processing of non-synesthesia-inducing shapes have been examined with EEG (Barnett et al., 2008) and fMRI (Sinke et al., 2012). Both studies found these processing differences to occur as early as in cortical area V1. Interestingly, the study by Barnett et al. (2008) showed that stimulus features, such as spatial frequency and contrast, led to significantly different early visual evoked potentials in synesthetes relative to controls. Specifically, high spatial-frequency Gabor-patches elicited an enhanced C1-component in synesthetes, which is generally attributed to processing in the primary visual cortex. Similarly, synesthetes were significantly more sensitive to the varying luminance contrast of checkerboard stimuli, showing enhanced P1-components over occipital regions bilaterally. These findings demonstrate that sensory processing of non-synesthesia-inducing stimuli occurs differently in the synesthetic brain, and could be attributed to altered circuitry in occipital areas. This raises two questions: (a) whether the sensory processing differences for non-synesthesia-inducing stimuli translate into a memory advantage, and (b) how the potentially subtle memory differences between synesthetes and controls can best be detected at the behavioral level.

To investigate the first question we developed a VAM test with achromatic pair-associates that differed in visual similarity. This manipulation aimed to tease out potential contributions of the synesthetes' early sensory and perceptual processing differences during associative learning and retrieval. To address the second question, we used a between-group design, comparing young synesthetes with young control participants and a third group of older adults who show characteristic, age-related deficits in perceptual processing (Fjell and Walhovd, 2004; Riis et al., 2009) and associative memory (Naveh-Benjamin, 2000). Comparing cognitive performance amongst three participant groups is an approach frequently used in neuropsychology to detect subtle memory differences, for example between older adults with questionable onset of dementia, healthy age-matched control participants, and patients with Alzheimer's disease (Fowler et al., 2002). A similar rationale was used in the present study: We expected the associative memory differences between young synesthetes and young controls to be too subtle to be detected for non-synesthesia-inducing stimuli, given that these stimuli are not known to evoke a conscious color experience in synesthetes to provide an advantage in perceptual processing over young adults. Thus, the inclusion of a third group of older adults provided another benchmark against which the other two groups could be compared. Specifically, we reasoned that the difference between young and older adults, vs. young synesthetes and older adults could uncover the synesthetes' subtle associative memory advantages. Intuitively, this would be similar to sampling from a larger range of points from the distribution of associative learning and memory ability, where synesthetes might be on the right of the mean (represented by young matched controls), and older adults might be on the left of the mean.

Compared to the emerging memory research in the synesthesia literature, VAM has been examined more extensively in older individuals. Age-related performance detriments are typically found during associative recognition (Naveh-Benjamin et al., 2004, 2007, 2009; Cowan et al., 2006; Cohn et al., 2008; Shing et al., 2008; Edmonds et al., 2012), as well as during encoding of visual pair-associates (Iidaka et al., 2001; Sperling et al., 2003). Associative memory deficits in older adults have been attributed to several neurological factors, such as white-matter hyper-intensities in memory-related fiber tracts (Lockhart et al., 2012), reduced gray-matter volume (Raz et al., 2005), and reduced activation in memory-related posterior parietal, inferior- and medial temporal lobe areas (Iidaka et al., 2001; Cabeza et al., 2004; Gutchess et al., 2005).

In the present study, we examined the effects of age and individual differences on associative encoding and associative retrieval. To this end, we employed a self-paced trial-and-error learning paradigm, in which participants were trained to performance criterion with a set of achromatic visual pair-associates (Learning phase). This learning paradigm was used to guarantee sufficient exposure to the pair-associates and satisfy subject-specific learning requirements. This allowed us to account for an age-related encoding deficit [(Naveh-Benjamin, 2000; Shing et al., 2010) for review] and to assess associative retrieval (Retrieval phase) after participants had reached the same performance level. The stimuli were black-and-white fractal pair-associates. These stimuli were chosen to prevent any advantageous primary or secondary color experiences for the synesthetes, therefore allowing us to investigate any potential generic VAM advantages in this group. Moreover, previous studies found that older adults, although generally impaired in VAM, show specific deficits in memory for abstract pair-associates (Iidaka et al., 2001). We therefore assumed that achromatic abstract stimuli would be most promising to elicit the relevant age- and individual differences in our study.

To tax the differential qualities of perception and memory between synesthetes and older adults, we further manipulated the ease with which the stimulus pairs could be associated during learning and discriminated from each other at retrieval. One effective way to manipulate associability/discriminability is by varying the picture similarity (Yago and Ishai, 2006; Poirier et al., 2012). Associative retrieval is less efficient if the visual similarity between cue and target decreases. Specifically, low similarity not only reduces the diagnostic value of the cue to its veridical target, but also increases competition among a range of other familiar images presented during retrieval, making the discriminability between matching and non-matching pair-associates more difficult. To exploit the differential effects of similarity during visual associative learning and retrieval in the present study, we chose a set of visually similar pair-associates that were expected to facilitate associability during learning and require less discriminability at retrieval (low memory load), and a set of visually dissimilar pair-associates that impede associability during learning and require high discriminability at retrieval (high memory load).

For the learning phase we hypothesized that, if the synesthetes' enhanced perceptual mechanisms for non-synesthesia inducing stimuli translated into an early learning advantage, this would emerge during encoding of similar pair-associates, which afford advantageous perceptual processing during associative learning. We examined pair-associative retrieval at two stages: immediately after the learning phase, and following a 30 min delay. At both retrieval stages, we derived signal detection measures of the Hit- and False alarm responses. We expected to find a memory advantage for similar over dissimilar pair-associates across groups and time of retrieval, due to their respective low and high demands of discriminability at test. Moreover, we hypothesized that if a retrieval advantage existed in synesthetes, a significant effect would emerge in the dissimilar condition that had the highest demands on discriminability.

## Learning phase

### Methods

#### Participants

Fourteen young non-synesthetes (8 female; age range = 19–29 years; *M* = 22.64), 14 older non-synesthetes (9 female; age range = 62–83 years; *M* = 68.79), and 14 young grapheme-color synesthetes (9 female; age range = 19–31 years; *M* = 22.50) took part in the experiment and were compensated for their time. All participants were healthy individuals with no history of any psychiatric, ophthalmological, or neurological diseases. Written informed consent was obtained from all participants. The study was approved by the BSMS Research Governance and Ethics committee. All groups were matched on the number of years of formal education (Young adults, *M* = 15.43 years, *SD* = 0.515; Older adults, *M* = 15.00 years, *SD* = 3.08; Synesthetes, *M* = 16.35 years, *SD* = 1.78), yielding no significant difference between groups, *F*_(2,39)_ = 1.558, *p* = 0.223.

Synesthetes were recruited from the University of Sussex and via the UK Synesthesia association website www.uksynaesthesia.com. All synesthetes reported seeing colors in response to letters or digits. To verify Synesthesia, we used the “Synesthesia battery” (Eagleman et al., 2007), available on www.synesthete.org, and the cut-off score of 1.43 (from Rothen et al., 2013). A mean consistency score of *M* = 0.84 (*SD* = 0.25) was obtained across the group of synesthetes, which confirmed their synesthesia.

We assessed all participants on three subtests of the object recognition test included in the Visual Object and Space Perception Battery [VOSP (Warrington and James, 1991)]. A summary of the participants' scores is provided in Table 1. A one-way between-subject (young adults, older adults, synesthetes) ANOVA on the averaged sum of the subtest scores revealed that there was no significant group difference in the performance of the object recognition test of the VOSP, *F*_(2, 39)_ = 0.032, *p* = 0.968, demonstrating that perceptual functions were comparable across groups.

**Table 1:** **Performance on the object recognition test of the Visual Object and Space Perception Battery (VOSP) (Warrington and James, 1991)**.

**Object recognition Subtests**	**Young adults**	**Older adults**	**Synesthetes**
	**(*N* = 14)**	**(*N* = 14)**	**(*N* = 14)**
	***M* (*SD*)**	***M* (*SD*)**	***M* (*SD*)**
Silhouettes (object naming)^a^	21.64 (3.27)	20.14 (3.95)	20.71 (4.00)
Object decision^b^	18.57 (0.85)	17.50 (2.10)	17.64 (1.82)
Progressive silhouettes^c^	7.79 (2.29)	10.71 (1.38)	9.75 (2.28)
Averaged sum of subtest scores	48.00 (4.27)	48.35 (5.40)	48.10 (4.63)

a *Maximum possible score is 30*.

b *Maximum possible score is 20*.

c *The lower the score, the better the performance*.

#### Stimuli

**Figure 1:**
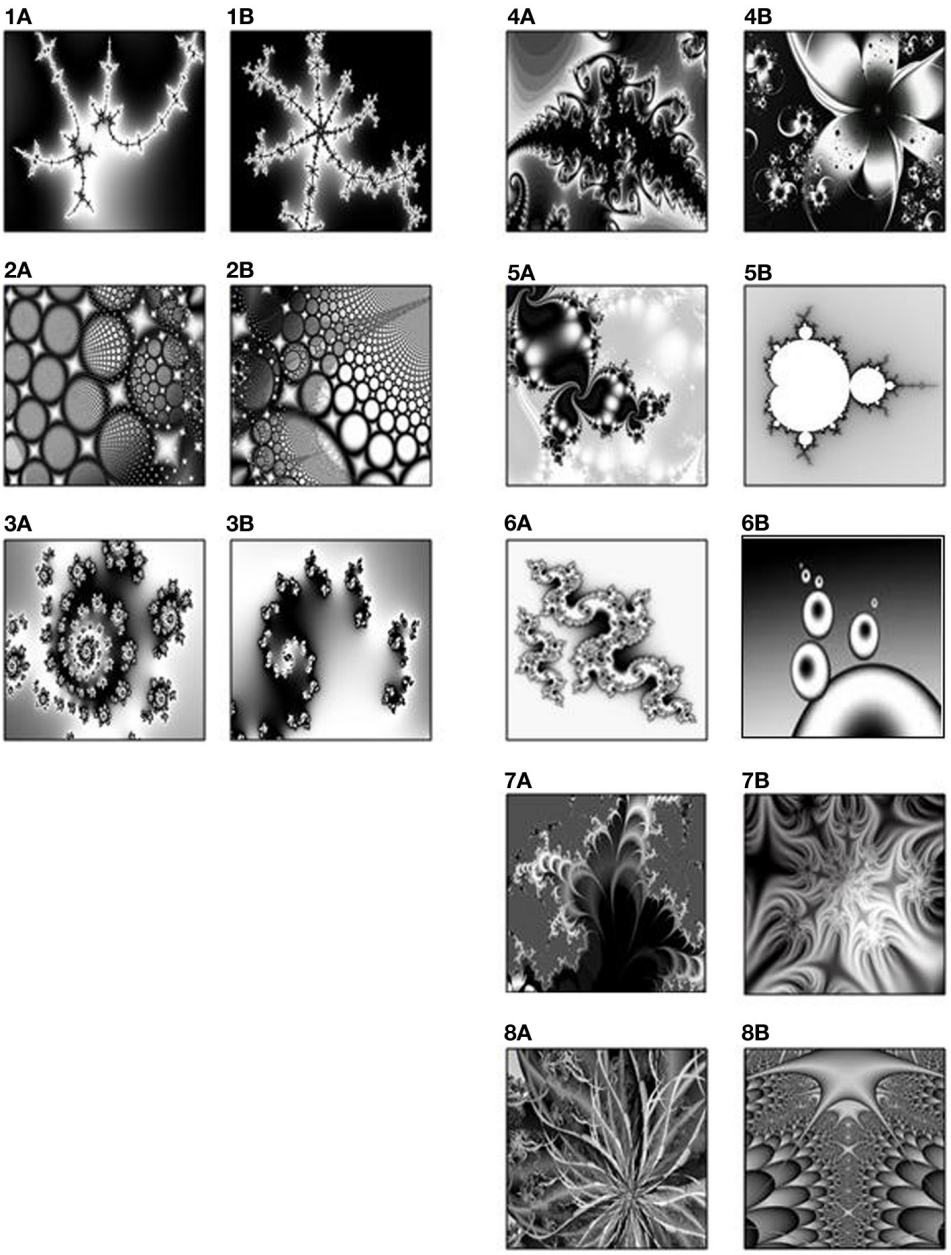
**The three similar pairs (1–3) on the left, and five dissimilar pairs (4–8) on the right, rated by an independent group of 19 participants**.

Eight pair-associates (black-and-white fractal images) (Figure 1) were selected from a pool of 18 pair-associates that had been rated for visual similarity by an independent group of 19 participants. Based on the mean-ratings of these 18 pairs of stimuli, we selected the five most dissimilar and the three most similar pairs. This ratio was chosen to compensate for the difference in their learning- and retrieval difficulty and to ensure successful memory across pair-associates. Associative learning and retrieval effects of the selected similar and dissimilar pair-associates were subsequently verified on another group of 15 young adults in a prior pilot experiment.

#### Procedure

**Figure 2:**
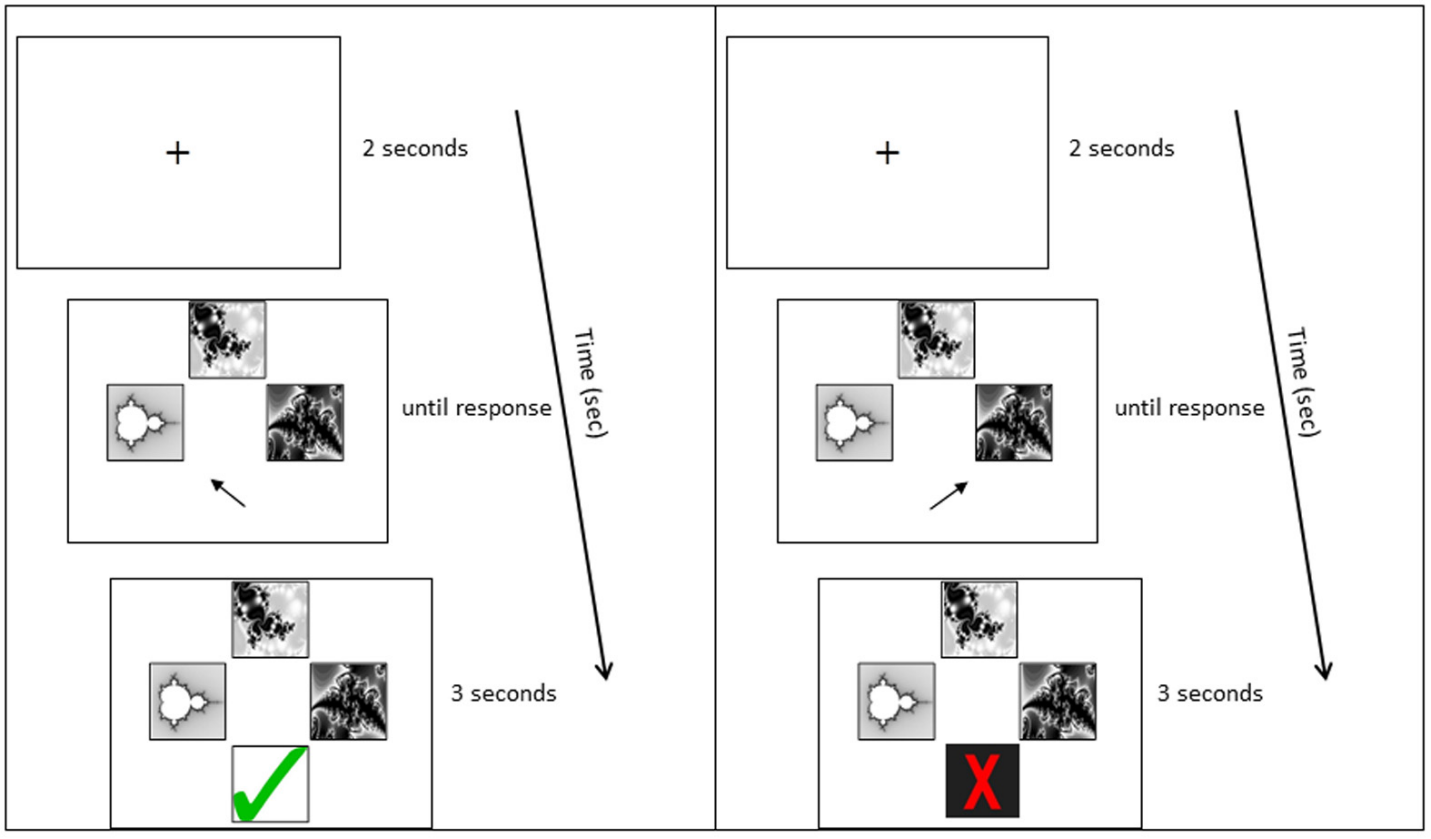
**Example learning trial**. Panels from top to bottom: Fixation cross; stimulus presentation; stimulus plus feedback. The left panel shows the feedback to a correct response; the right panel shows the feedback to an incorrect response.

A computer-based task was developed for pair-associative learning. Participants were seated in front of a 19 inch computer monitor, at a distance of 60 cm; the stimuli subtended approximately 3° of visual angle. Participants were asked to learn the correct combination of eight pair-associates via trial-and-error. They were instructed to memorize the pair-associates for a subsequent memory test. Each trial began with a fixation cross (2 s), followed by a cue picture presented at the top of the screen and two possible matching target pictures below (Figure 2). The non-matching target was one from the set of pair-associates to be learned, rather than of a novel shape, to ensure equal picture familiarity. Participants were asked to indicate which of the two target pictures belonged with the cue, by pressing the left or right arrow key. The pictures stayed on screen until a response was recorded. Following the response, visual feedback appeared below the pictures (3 s), indicating whether the matching target had been identified correctly or not (green tick or red cross, respectively). Cue and target shapes of all pair-associates were presented interchangeably during learning: a stimulus that had been presented as the cue in one Run constituted the target in the following Run. A minimum of two Runs was required in the learning phase. Each Run contained eight trials and participants performed the test until they achieved a minimum of seven out of eight Hits on two successive Runs (learning criterion). Stimuli were delivered using Presentation® 14.9 (Neurobiobehavioral Systems, Inc.).

### Data analysis

#### Effect sizes

Cohen's *d* was used as an effect size measure for all pair-wise *post-hoc* comparisons. The following formula was used for calculation: *d* = *m*1 − *m*2/σ, where *m*1 = mean of group1, *m*2 = mean of group2, σ = the pooled standard deviation of the group means (Cohen, 1988). Cohen's *d* can be interpreted as: *d* = 0.20 (small effect); *d* = 0.50 (medium effect) and *d* = 0.80 (large effect; Cohen, 1992).

Partial eta squared (η^2^_*p*_) was used as an effect size measure in all analyses of variance (ANOVA) and in all analyses of covariance (ANCOVA). η^2^_*p*_ was calculated using the formula: η^2^_*p*_ = SS_effect_/SS_effect_+ SS_residual_, where SS_effect_ = the sum of squares for the effect of interest and SS_residual_ = the sum of squares of the error associated with the effect of interest. η^2^_*p*_ provides the effect of “the proportion of variance that a variable explains that is not explained by other variables in the analysis” (Field, 2009; p. 415) and can be interpreted as: η^2^_*p*_ = 0.01 (small effect); η^2^_*p*_ = 0.06 (medium effect) and η^2^_*p*_ = 0.14 [large effect; (Cohen, 1988)].

#### Power analysis

Given the relatively small sample sizes in our three groups, we calculated the achieved power in all pair-wise *post-hoc* comparisons to supplement our null hypothesis significance tests. The power calculations were performed using the G^*^Power calculator v. 3.1.6. (Faul et al., 2009).

### Results

#### Pair-associative learning

**Figure 3:**
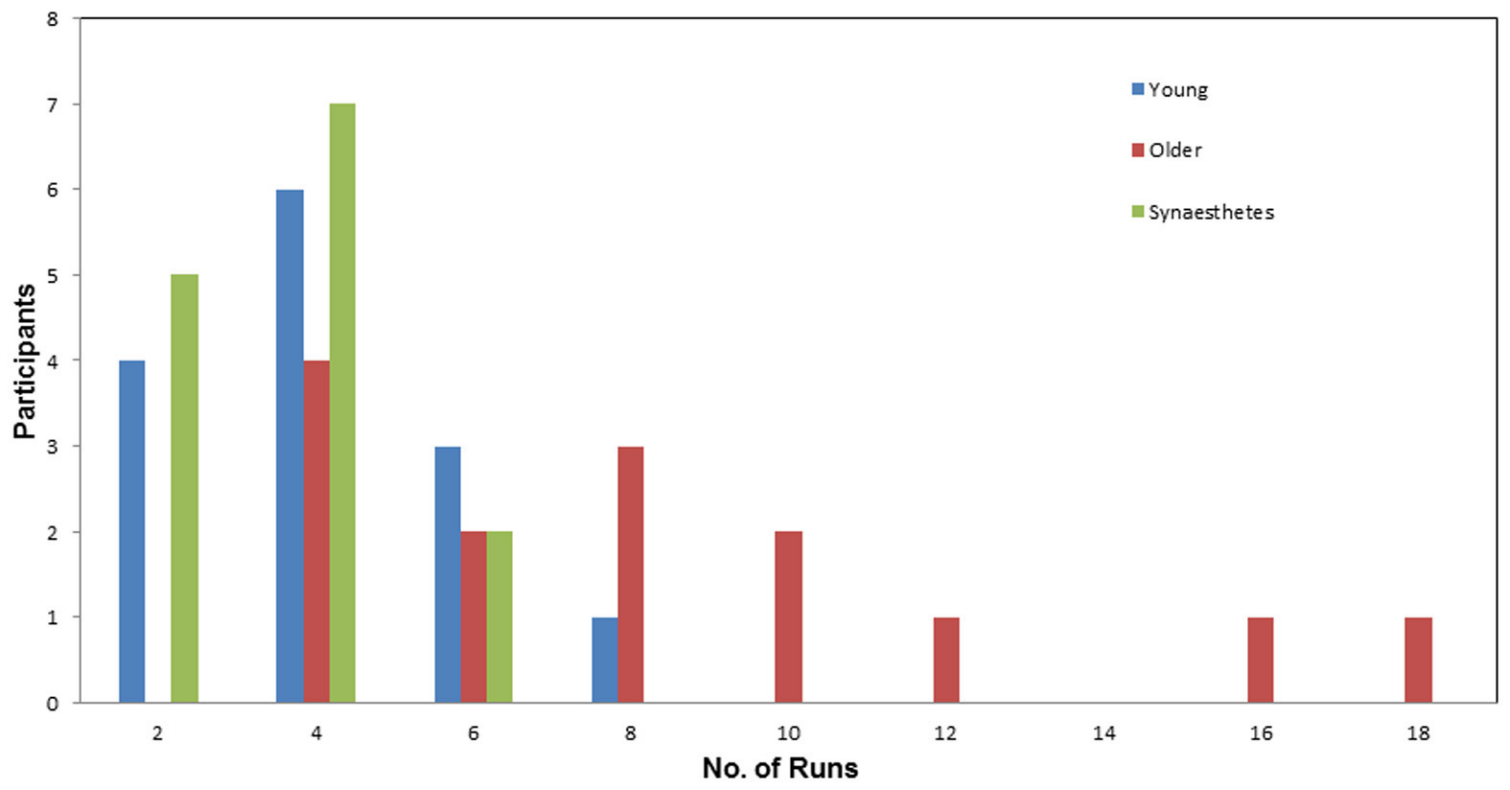
**Number of runs required by participants to learn the pair-associates to criterion**. Average number of runs for the young (*M* = 3.64), for the synesthetes (*M* = 3.21), and for the older adults (*M* = 9.93). The young adults and the synesthetes learned significantly faster than the older adults.

***Number of runs***. Figure 3 illustrates the number of Runs required by each participant to learn the full set of eight pair-associates (similar and dissimilar pairs) to criterion. The average number of Runs was greatest for the older adults (*M* = 7.93; *SE* = 1.23), followed by young adults (*M* = 3.64; *SE* = 0.48) and fewest for the synesthetes (*M* = 3.21; *SE* = 0.30). A One-Way ANOVA, with group (young adults, older adults, synesthetes) as the between-subject factor, yielded a significant effect on the number of Runs [*F*_(2, 39)_ = 11.16, *p* < 0.001]. Subsequent Tukey (HSD) *post-hoc* comparisons revealed significant learning differences between synesthetes and older adults (*p* < 0.001; *d* = 1.47; power = 0.58), young and older adults (*p* = 0.001; *d* = 1.28; power = 0.40), while there was no significant difference between synesthetes and young adults (*p* = 0.920; *d* = 0.29; power = 0.94).

***Similarity effects on pair-associative learning***. To examine the group differences in learning the similar and dissimilar pair-associates, two ANCOVA were performed. For these analyses, each participant's trial-by-trial responses were averaged across the total number of Runs for each condition and were entered as the dependent variable. Group (young adults, older adults, synesthetes) was included as the fixed effect and the total number of Runs was entered as the covariate.

Next, we examined whether there were any group differences in the successive learning rate of similar and dissimilar pair-associates over the first five Runs (the maximum number of Runs required by the synesthetes). To this end, we performed five One-Way ANOVA's per condition (similar, dissimilar), with group as the between-subject factor. In these analyses, we successively averaged the Hit-rate over an increasing number of Runs. In other words, we analyzed the variance of the cumulative Hit-rates between groups over the first five Runs to examine if and when a significant group effect would emerge.

***Similar pairs***. Learning the similar pair-associates yielded high Hit-rates (averaged across all Runs) in all three groups [young (*M* = 96.87; *SE* = 1.40), older adults (*M* = 91.23; *SE* = 3.83), and synesthetes (*M* = 98.93; *SE* = 0.73)]. The ANCOVA revealed that the covariate (number of Runs) did not significantly predict Hit-rate, *F*_(1, 38)_ = 2.473, *p* = 0.124, η^2^_*p*_ = 0.061. Moreover, there was no significant group effect on the averaged Hit-rate, irrespective of whether the effect of the covariate was removed, *F*_(2, 38)_ = 0.530, *p* = 0.593, η^2^_*p*_ = 0.027, or not, *F*_(2, 39)_ = 2.78; *p* = 0.074; η^2^_*p*_ = 0.125.

**Figure 4:**
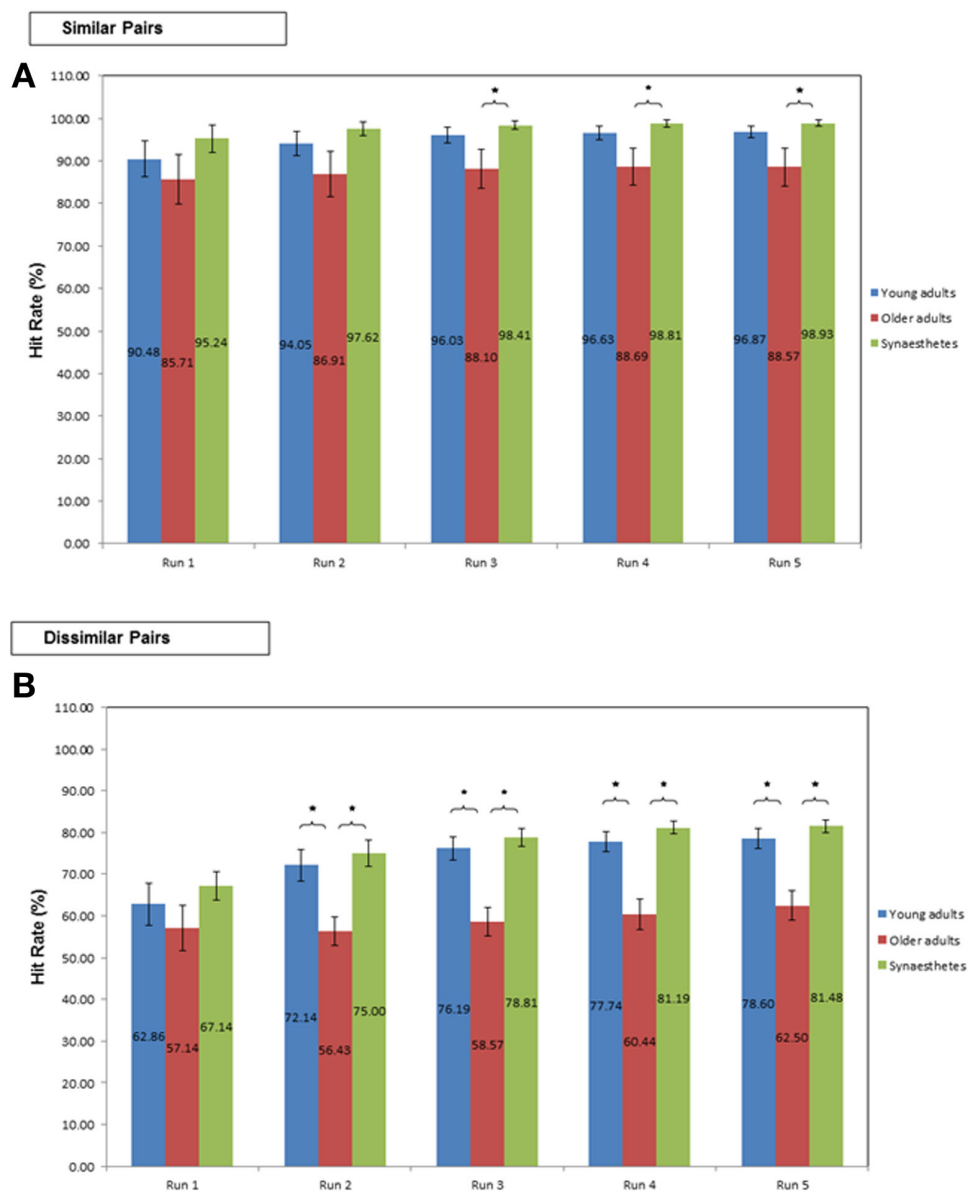
**Percent Hit-rate during learning in young adults, older adults and synesthetes**. Learning of **(A)** similar pair-associates, and **(B)** dissimilar pair-associates illustrated on the first five Runs. Error bars: standard error of the mean. Significance level: ^*^p ≤ 0.05.

As shown in Figure 4A, the two One-Way ANOVA's of the first two Runs yielded no significant group effect on the cumulative Hit-rate (both *p* > 0.05). Starting on the third Run however, the group effect was significant [*F*_(2, 39)_ = 3.01, *p* = 0.043]. Tukey (HSD) *post-hoc* comparisons revealed that synesthetes performed significantly better than older adults (*p* = 0.044), yielding a large effect size of *d* = 0.86 but insufficient power (0.57). No significant difference was found between young and older adults (*p* = 0.147; *d* = 0.63; power = 0.57) or between young adults and synesthetes (*p* = 0.834; *d* = 0.43; power = 0.91).

Similarly, in Runs 4 and 5, we found a significant group effect on the cumulative Hit-rate [Run 4: *F*_(2, 39)_ = 4.04, *p* = 0.025; Run 5: *F*_(2, 39)_ = 4.05, *p* = 0.025]. In both Runs, synesthetes performed significantly better than older adults (Run 4: *p* = 0.027; Run 5: *p* = 0.028), yielding large effect sizes (Run 4: *d* = 0.92; Run 5: *d* = 0.9), but insufficient power (Run 4: power = 0.54; Run 5: power = 0.53). No significant difference was found between young and older adults (Run 4: *p* = 0.099; Run 5:*p* = 0.092), coupled with medium effect sizes (Run 4: *d* = 0.69; Run 5: *d* = 0.7) and insufficient power (Run 4: power = 0.55; Run 5: power = 0.55). The difference between young adults and synesthetes was non-significant (Run 4: *p* = 0.830; Run 5: *p* = 0.854), however, the effect size measures were medium (Run 4: *d* = 0.49; Run 5: *d* = 0.51) and the statistical power was high (Run 4: power = 0.93; Run 5: power = 0.93).

***Dissimilar pairs***. The averaged Hit-rate across all Runs in the dissimilar pair-learning condition was highest in the synesthetes (*M* = 81.48; *SE* = 1.54), followed by young (*M* = 79.45; *SE* = 1.90), and older adults (*M* = 67.22; *SE* = 2.53). The ANCOVA revealed that the covariate (number of Runs) made a significant contribution to the Hit-rate, *F*_(1,38)_ = 16.869, *p* < 0.001, η^2^_*p*_ = 0.307. With the effect of the number of Runs removed, there was a significant group effect on the averaged Hit-rate *F*_(2, 38)_ = 3.419, *p* = 0.043, η^2^_*p*_ = 0.153. Tukey *post-hoc* comparisons revealed a significant difference between synesthetes and older adults (*p* = 0.015, *d* = 1.89; power = 0.99), as well as between young and older adults (*p* = 0.041, *d* = 1.52; power = 0.97). The difference between synesthetes and young adults was not significant (*p* = 0.566, *d* = 0.33; power = 0.69).

As shown in Figure 4B, the One-Way ANOVA of the first Run in the dissimilar condition yielded no significant group effect on the cumulative Hit-rate [*F*_(2, 39)_ = 1.12, *p* = 0.336]. Starting on the second Run however, there was a significant group effect on Hit-rate [*F*_(2, 39)_ = 8.39, *p* = 0.001]. Tukey (HSD) *post-hoc* comparisons showed a significantly greater Hit-rate for synesthetes relative to older adults (*p* = 0.001, *d* = 1.58; power = 0.68) and for young adults relative to older adults (*p* = 0.007, *d* = 1.21; power = 0.61), while the difference between young adults and synesthetes was not significant (*p* = 0.829, *d* = 0.23; power = 0.86). The significant group effect on the cumulative Hit-rate was maintained throughout Runs 3–5 [Run 3: *F*_(2, 39)_ = 15.10, *p* < 0.001; Run 4:*F*_(2, 39)_ = 17.66, *p* < 0.001; Run 5: *F*_(2, 39)_ = 15.67, *p* < 0.001]. Specifically, for Runs 3–5, Tukey (HSD) *post-hoc* comparisons revealed that both groups, synesthetes and young adults, performed significantly better than older adults (both groups, Runs 3–5: *p* < 0.001), while there was no significant difference between young adults and synesthetes (Runs 3–5: *p* > 0.05). Interestingly, although the effect sizes for the comparison of synesthetes and older adults, and for young and older adults were large (Runs 3–5, *d* > 1.5), we only obtained sufficient power for the comparison of synesthetes and older adults (Run 3: power = 0.91; Run 4: power = 0.95; Run 5: power = 0.91), while the comparison of young and older adults was underpowered (Run 3: power = 0.67; Run 4: power = 0.67; Run 5: power = 0.61). For the comparison of young adults and synesthetes we found a small effect size in Run 3 (*d* = 0.29), followed by a medium effect size in Runs 4 (*d* = 0.48) and 5 (*d* = 0.41). Sufficient power for these effects were obtained throughout Runs 3–5 (power > 0.80).

### Discussion

The results of the learning phase demonstrated two major points. First, interrogating different measures of associative learning (e.g., number of Runs vs. averaged Hit-rate vs. cumulative Hit-rate) is critical in establishing the precise group differences. Second, supplementing conventional null hypothesis significance testing with power analyses is crucial for small group sizes to be able to make inferences about the reliability of the obtained alpha-values and effect size measures.

The first point is illustrated by the analyses of the number of Runs (representing the crudest measure of group differences in associative learning) and of the averaged Hit-rate in the dissimilar condition. Both results suggest an effect of age on associative learning, with no effect of synesthesia over and above age. Moreover, the averaged Hit-rate in the similar condition, which was high and comparable across groups, suggested a generic benefit of similarity in associative learning (Poirier et al., 2012), but no specific effect of synesthesia.

The more interesting relationships could only be observed after interrogating cumulative Hit-rates. In the similar condition, the results of the null hypothesis significance tests were in line with our hypothesis, suggesting that synesthetes showed an associative learning advantage, which could only be detected relative to older adults. The fact that the young adults showed no significant learning advantage relative to older adults rules out a mere age-effect for synesthetes (who were age-matched to the young adults), and instead suggests an additive effect of synesthesia and perceptual similarity on associative learning. The argument is strengthened by effect size measures, showing that the difference between young and older adults was medium, while for synesthetes and older adults it was large. However, the results of the power analyses suggest that there is only a 50–60% chance of replicating the findings. Thus, the observed group differences in the similar condition, although detected in our present sample, cannot be extrapolated to the wider population. Interestingly, we also found a medium effect size between young adults and synesthetes, despite the non-significant differences between these groups, indicating that there was a meaningful performance advantage of synesthetes over young adults. Nevertheless, given that the achieved power in this comparison was above 90%, we are safe in retaining the null hypothesis to avoid conducting a Type I error (Cohen, 1992). In summary, our sample of 14 synesthetes demonstrated an enhanced sensitivity to perceptual similarity relative to the 14 older adults. Previous studies have shown the synesthetes' differential processing mechanisms of non-synesthesia-inducing stimuli at the perceptual level (Barnett et al., 2008; Sinke et al., 2012). Our results replicate and extend these findings, by showing a performance gain for synesthetes during learning of similar pair-associates.

In the dissimilar condition, the results of the cumulative Hit-rate analysis showed a significant learning advantage for synesthetes and young adults relative to older adults. However, although the effect size measures were large in both comparisons, only the comparison of synesthetes and older adults yielded enough power (above 90%) for the findings to be reliable. Thus, the results suggest a reliable learning advantage in synesthetes for non-synesthesia inducing, dissimilar pair-associates, which could only be detected against older adults. The difference between synesthetes and young adults was non-significant, however, the parametric increase in effect size measures (from small to medium) from Runs 2–4, demonstrates that the size of the difference between synesthetes and young adults became increasingly larger over time.

## Retrieval phase

### Methods

#### Participants

We tested the same participants as in the learning phase.

#### Procedure

**Figure 5:**
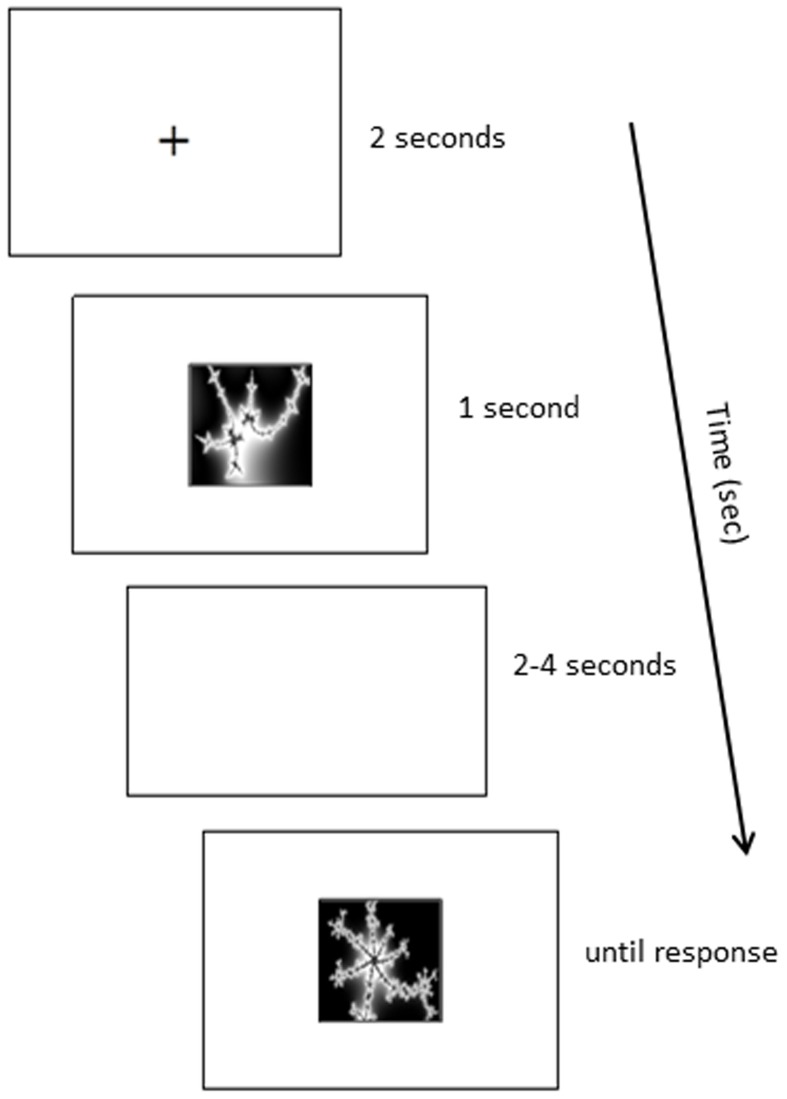
**Example retrieval trial**.

Participants remained seated in front of the computer monitor to take part in the immediate retrieval test. They were informed that they would be tested on the eight pair-associates acquired during the learning phase. Each trial began with a fixation cross (2 s), followed by a cue picture presented at the center of the screen (1 s). Participants were asked to use the cue to recall the matching pair-associate. Next, a blank white screen was shown for a variable delay of 2–4 s, during which participants had to hold the matching picture in mind. Then, a target appeared, which was either the matching stimulus to the cue, or another picture randomly chosen from the learned set of pair-associates (non-match). The target remained on screen until participants pressed a key, indicating whether it was a match or not. Figure 5 presents an example of such a trial. Participants' retrieval performance was assessed on two Runs. Each Run contained 16 trials, including eight match trials and eight non-match trials that were randomly interleaved. The paired stimuli were presented interchangeably as cues or targets across the two Runs. No feedback was provided on the accuracy of the participants' responses.

Following a 30 min delay, during which participants carried out the object recognition test of the VOSP (Warrington and James, 1991), a surprise second retrieval test was administered. The procedure for this delayed retrieval test was identical to the immediate retrieval task described above.

At the end of the experiment the synesthetes were asked whether they had perceived colors in response to the visual pair-associates during the learning and/or retrieval phase. None of the synesthetes reported any color experiences.

### Data analysis

#### Signal detection

We carried out a signal detection analysis, deriving measures of *d* prime (*d*′) and criterion *C* (Stanislaw and Todorov, 1999). Measures of *d*′ represent a person's sensitivity in discriminating between signal trials (matching pair-associates) and noise trials (non-matching pair-associates). Thus, *d*′ returns the difference between an individual's probability to give positive responses to matching pair-associates (Hits) and the probability of giving positive responses to non-matching pair-associates also (False alarms), providing a standardized estimate of effective memory retention (see e.g., Cowan et al., 2006; Cohn et al., 2008). Furthermore, we calculated the signal detection criterion C, which is a measure of response bias. A low subjective threshold for signal detection will lead to a bias toward “yes” responses for matching and non-matching pair-associates, and is expressed by negative scores of *C*. Biased responses can mask participants' sensitivity in discriminating between signal and noise trials and lead to incorrect assumptions about their memory.

D prime and criterion *C* were calculated as follows: all probability scores of Hits_similar_ and False alarms_similar_ (respectively: Hits_dissimilar_ and False alarms_dissimilar_) were converted into *z* scores using the inverse phi function [Φ^−1^ (probability)] (Stanislaw and Todorov, 1999). To enable the conversion, all False alarm rates of 0 were raised to 0.01; all Hit-rates of 1 were lowered to 0.99 (Cowan et al., 2006). For *d*′, the *z* scores of False alarms were subtracted from the *z* scores of Hits according to the following formulae:

$$\begin{array}{l} d^{\prime }=\Phi^{-1}({\text{Hits}}_{\text{similar}})-\Phi^{-1}(\text{False }{\text{alarms}}_{\text{similar}})\\ d^{\prime }=\Phi^{-1}({\text{Hits}}_{\text{dissimilar}})-\Phi^{-1}(\text{False }{\text{alarms}}_{\text{dissimilar}}). \end{array}$$

Measures of criterion *C* were obtained using the following formulae:

$$\begin{array}{l} C=-\Phi^{-1}({\text{Hits}}_{\text{similar}})+\Phi^{-1}(\text{False }{\text{alarms}}_{\text{similar}})/2\\ C=-\Phi^{-1}({\text{Hits}}_{\text{dissimilar}})+\Phi^{-1}(\text{False }{\text{alarms}}_{\text{dissimilar}})/2. \end{array}$$

### Results

#### D prime

**Figure 6:**
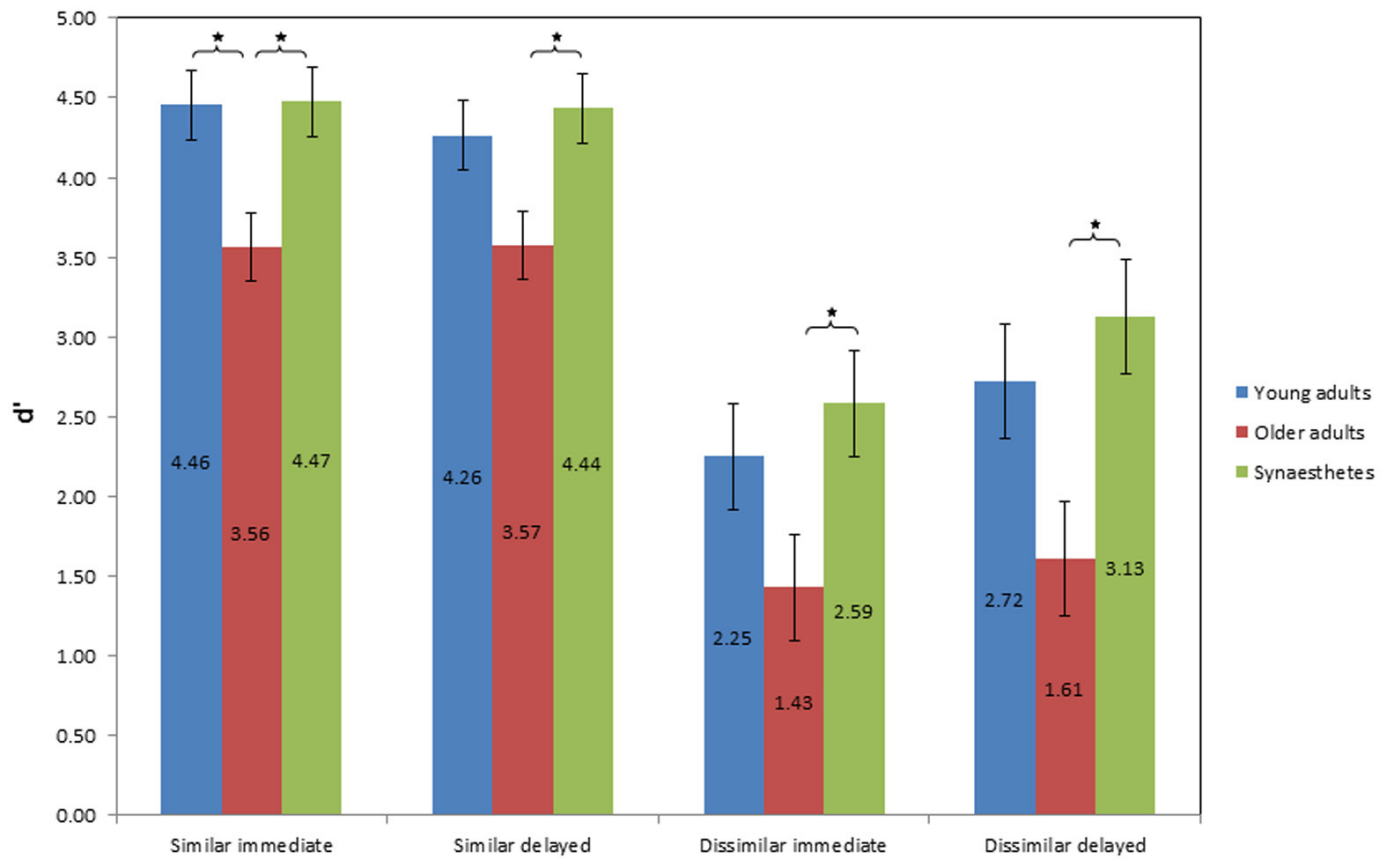
**Values of mean *d* prime score of sensitivity as a function of group, condition, and time of retrieval**. Error bars: standard error of the mean. Higher *d*' scores represent greater sensitivity in discriminating between matching and non-matching pair-associates, indicating higher effective memory retention. Significance level: ^*^p ≤ 0.05.

Figure 6 illustrates the mean *d* prime scores of sensitivity as a function of group, similarity of pair-associates and time of retrieval. A 3 × 2 × 2 mixed factorial ANOVA was conducted, with group (young adults, older adults, synesthetes) as the between-subject factor, condition (similar, dissimilar) and time of retrieval (immediate, delayed) as within-subject factors. We found a significant main effect of group on sensitivity (across similar and dissimilar pair-associates), *F*_(2, 39)_ = 9.088, *p* = 0.001, η^2^_*p*_ = 0.318. Tukey (HSD) *post-hoc* comparisons revealed that the difference in sensitivity was found between young and older adults, *p* = 0.008, *d* = 0.83; power = 0.27, between synesthetes and older adults, *p* = 0.001, *d* = 1.12; power = 0.26, but not between young adults and synesthetes, *p* = 0.679, *d* = 0.26; power = 0.74.

There was also a significant main effect of similarity on sensitivity, *F*_(1, 39)_ = 106.725, *p* < 0.001, η^2^_*p*_ = 0.732, suggesting that the *d* prime scores differed between the similar and dissimilar condition. The interaction between similarity and group was not significant, *F*_(2, 39)_ = 0.541, *p* = 0.587, η^2^_*p*_ = 0.027.

No significant main effect on sensitivity was found for time of retrieval, *F*_(1, 39)_ = 1.740, *p* = 0.195, η^2^_*p*_ = 0.043. However, there was a near-significant interaction between similarity and time of retrieval, *F*_(1, 39)_ = 3.847, *p* = 0.057, η^2^_*p*_ = 0.090, suggesting that although sensitivity was affected by the similarity of the pair-associates, this differed according to the time of retrieval. Figure 6 illustrates that while sensitivity in the similar condition was comparable across time, it was enhanced at delayed retrieval in the dissimilar condition. No interaction effect was found between time of retrieval and group, *F*_(2, 39)_ = 0.143, *p* = 0.867, η^2^_*p*_ = 0.007, or between condition, time of retrieval and group, *F*_(2, 39)_ = 0.402, *p* = 0.672, η^2^_*p*_ = 0.020.

In the following sections, we assessed the group effects on sensitivity further. To this end, we carried out four One-Way ANOVA's, using group as the fixed effect, and the four respective conditions as the dependent variables (Similar_immediate_; Similar_delayed_; and Dissimilar_immediate_; Dissimilar_delayed_).

#### D prime of similar pair retrieval

Figure 6 shows the average *d* prime scores of sensitivity for immediate and delayed retrieval of similar pair-associates. The two One-Way ANOVA's for the similar condition yielded a significant effect of group on sensitivity at both retrieval stages [immediate: *F*_(2, 39)_ = 5.712; *p* = 0.007; delayed: *F*_(2, 39)_ = 4.394; *p* = 0.019]. Tukey (HSD) *post-hoc* comparisons for immediate retrieval showed that while synesthetes and young adults did not differ from each other (*p* = 0.998, *d* = 0.04, power = 0.99), synesthetes and older adults did (*p* = 0.014, *d* = 1.02, power = 0.53), as did young and older adults (*p* = 0.016, *d* = 0.98, power = 0.52).

At delayed retrieval, there was no significant difference between synesthetes and young adults (*p* = 0.843, *d* = 0.23, power = 0.87), and young and older adults (*p* = 0.076, *d* = 0.78, power = 0.59), while the synesthetes maintained a significant retrieval advantage over older adults (*p* = 0.021, *d* = 1.01, power = 0.59).

#### D prime of dissimilar pair retrieval

Figure 6 shows the average *d* prime scores of sensitivity for immediate and delayed retrieval of dissimilar pair-associates. The One-Way ANOVA at immediate retrieval yielded a near-significant effect of group on sensitivity [*F*_(2, 39)_ = 3.19; *p* = 0.052]. Tukey (HSD) *post-hoc* comparisons revealed that the effect was driven by the synesthetes, whose *d*' scores were significantly above those of older adults (*p* = 0.048), yielding a large effect size of *d* = 1.08 and sufficient power (0.78), whereas we found no difference between young and older adults (*p* = 0.202), with a medium effect (*d* = 0.64) and insufficient power (0.65), or between synesthetes and young adults (*p* = 0.758), showing a small effect of *d* = 0.27 and sufficient power (0.81).

Likewise, at delayed retrieval, we found a significant effect of group on sensitivity [*F*_(2, 39)_ = 4.7; *p* = 0.014]. Tukey (HSD) *post-hoc* comparisons again revealed a significant difference between synesthetes and older adults (*p* = 0.013), with a large effect size (*d* = 1.23), but with reduced power (0.72) relative to the immediate condition, while the difference between young and older adults was not significant (*p* = 0.083), albeit showing a large effect size of *d* = 0.87, but insufficient power (0.69). No significant difference was found between synesthetes and young adults (*p* = 0.708, *d* = 0.3, power = 0.78). Thus, across two time points, we found evidence for a subtle memory advantage in synesthetes for dissimilar pair-associates, which emerged in comparison to older adults.

#### Criterion C

**Figure 7:**
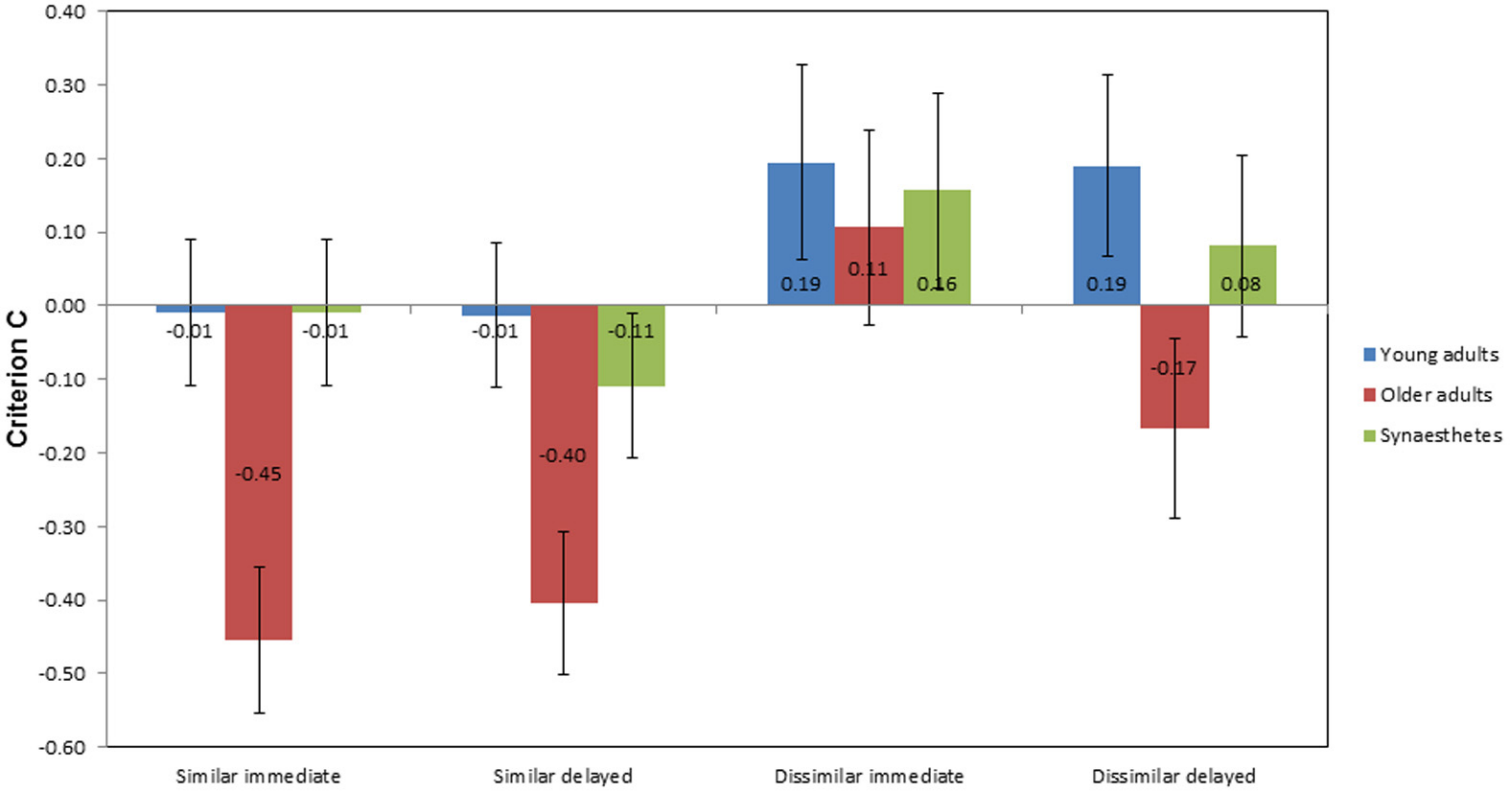
**Mean criterion *C* scores as a function of group, condition, and time of retrieval**. Negative scores indicate a bias toward “yes” responses for matching and non-matching pair-associates, while positive scores indicate a bias toward “no” responses.

Figure 7 illustrates the mean scores of criterion *C* as a function of group, condition, and time of retrieval. In the similar condition, older adults showed the largest negative scores across groups at immediate (*M* = −0.45; *SE* = 0.14) and delayed retrieval (*M* = −0.40; *SE* = 0.11), indicating a bias toward “yes” responses. A negligible response bias toward yes responses was found for the young adults and the synesthetes at immediate retrieval (both *M* = −0.01; *SE* = 0.07). At delayed retrieval, we found a decrease in the synesthetes' criterion *C* (*M* = −0.11; *SE* = 0.074), with no change in the young adults (*M* = −0.01; *SE* = 0.10). In the dissimilar condition, we found a bias toward “no” responses across groups at immediate retrieval, as indicated by positive values of *C* (young adults: *M* = 0.19; *SE* = 0.13; older adults: *M* = 0.11; *SE* = 0.15; synesthetes: *M* = 0.16; *SE* = 0.12). At delayed retrieval, biased “no” responses were found for young adults (*M* = 0.19; *SE* = 0.14) and synesthetes (*M* = 0.08; *SE* = 0.12), while older adults tended to be biased toward giving “yes” responses (*M* = −0.17; *SE* = 0.11).

A 3 × 2 × 2 mixed factorial ANOVA was performed, with group as the between-subject factor, condition (similar, dissimilar), and time of retrieval (immediate, delayed) as within-subject factors. We found a significant main effect of group on criterion bias, *F*_(2, 39)_ = 5.590, *p* = 0.007, η^2^_*p*_ = 0.223. Tukey (HSD) *post-hoc* comparisons revealed that the difference in criterion bias was significant between young and older adults, *p* = 0.009, *d* = 0.75, power = 0.22, between synesthetes and older adults, *p* = 0.038, *d* = 0.64, power = 0.33, but not between young adults and synesthetes, *p* = 0.823, *d* = 0.16, power = 0.84.

There was also a significant main effect of similarity on criterion bias, *F*_(1, 39)_ = 23.004, *p* < 0.001, η^2^_*p*_ = 0.371, suggesting that the biased responses differed between the similar and dissimilar condition. As can be seen in Figure 7, participants tended to give more biased “yes” responses in the similar condition, whilst providing more hesitant “no” responses in the dissimilar condition. However, the interaction between similarity and group was not significant, *F*_(2, 39)_ = 1.657, *p* = 0.204, η^2^_*p*_ = 0.078.

No significant main effect on criterion bias was found for time of retrieval, *F*_(1, 39)_ = 0.991, *p* = 0.326, η^2^_*p*_ = 0.025 and there was no interaction between time of retrieval and group, *F*_(2, 39)_ = 0.231, *p* = 0.795, η^2^_*p*_ = 0.012. Moreover, there was no significant interaction between similarity and time of retrieval, *F*_(1, 39)_ = 0.850, *p* = 0.362, η^2^_*p*_ = 0.021, or between similarity, time of retrieval and group, *F*_(2, 39)_ = 1.060, *p* = 0.356, η^2^_*p*_ = 0.052.

## Discussion

In line with our first hypothesis, the retrieval results of the 3 × 2 × 2 ANOVA demonstrated that the stimulus similarity manipulation was effective at influencing associative retrieval, as shown by significantly higher *d* prime scores during retrieval of similar compared to dissimilar pair-associates. These results replicate previous findings by Poirier et al. (2012), suggesting that reduced similarity between a cue and a target increases the demands of discriminability, not only within, but also between pair-associates. However, the *d* prime scores of dissimilar pairs were higher in the delayed than in the immediate condition, yielding a near-significant interaction between similarity and time of retrieval. One likely explanation for this result is an effect of practice.

We further predicted that if a retrieval advantage existed in synesthetes, a significant effect would emerge in the dissimilar condition that had the highest demands on discriminability. This was supported by the results of the two One-Way ANOVA's of the dissimilar condition, at immediate and delayed retrieval. Specifically, in these two ANOVA's, we found that synesthetes performed significantly better than older adults, and the results were coupled with large effect sizes. More importantly, the results demonstrated sufficient power to be reliable, especially in the immediate retrieval condition. Thus, our retrieval results corroborate the notion of a memory advantage in synesthetes for non-synesthesia inducing stimuli, which emerged during dissimilar pair learning, and which could only be detected against older adults.

The fact that the comparisons between young and older adults in the two dissimilar conditions were non-significant but underpowered suggests that with increased sample sizes we might have observed a significant retrieval advantage of young relative to older adults. This may be particularly pertinent in the dissimilar delayed retrieval condition, where the alpha value between young and older adults reached 0.083, coupled with a large effect size. However, given the likely carry-over effects from immediate retrieval (see interaction between similarity and time of retrieval), the results of the delayed retrieval condition may be confounded by these effects. We therefore argue that the results of the dissimilar immediate retrieval condition provide a more accurate measure of associative memory.

Indeed, the non-significant result between young and older adults in the dissimilar condition is rather atypical in the recognition memory literature, where poorer associative memory performance in older adults is the norm (Sperling et al., 2003; Naveh-Benjamin et al., 2004, 2009; Cohn et al., 2008; Edmonds et al., 2012). We attribute this finding to the effects of the self-paced learning paradigm used in learning phase. These results are encouraging, as they suggest that when older adults are given sufficient time to learn visual pair-associates, their associative retrieval becomes non-significantly different from that of young adults. Implications of this finding are discussed further in the General Discussion.

With respect to the similar retrieval condition, significance testing suggested a subtle memory advantage for similar pair-associates in synesthetes, which could only be detected against older adults (at delayed retrieval), and which was not found for the comparison of young and older adults. However, the power analyses revealed that both comparisons, that of synesthetes and young adults relative to older adults, were not reliable, and that the only result showing high power was the non-significant comparison of young adults relative to synesthetes. These findings demonstrate that the similar pair-associates were highly associable, which made it difficult to establish significant and reliable memory differences between groups, even with older adults.

While previous associative memory studies tended to investigate age-related changes in sensitivity (Cowan et al., 2006; Cohn et al., 2008; Naveh-Benjamin et al., 2009), few studies have measured participants' criterion bias (but see Cowan et al., 2006). Given the heterogeneous participant groups tested in the present study, it was deemed important to include measures of bias. Our findings showed that older adults were biased toward giving “yes” responses throughout the similar and dissimilar conditions at delayed retrieval. One possibility for the biased responses might be the older adults' proclivity to rely on picture familiarity (Naveh-Benjamin et al., 2009; Edmonds et al., 2012). Especially in the case of similar pair-retrieval, where familiarity is easily established, this would trigger feelings of knowing the answer following the presentation of a cue, thus biasing older adults to provide positive responses irrespective of target-compatibility. The effect of increased familiarity was also evident in the dissimilar condition, where older adults were first biased toward giving “no” responses at immediate retrieval, but were the only group to provide “yes” responses at delayed retrieval, after the familiarity of the stimuli increased. Importantly, reliance on familiarity (rather than actual discriminability) has been explained by the reduced neural selectivity found in older adults' inferior temporal cortex, which alters perceptual sensitivity and spurs biased responses toward familiarity (Park et al., 2004). A similar explanation can account for the slight bias toward “yes” responses in synesthetes that we found in the similar condition at delayed retrieval. Synesthetes were previously found to have enhanced neuronal excitability in the primary visual cortex, which lowered the signal-to-noise ratio of their conscious synesthetic experiences (Terhune et al., 2011). These lower thresholds of cortical excitability in synesthetes may have spurred biased responses toward relying on familiarity heuristics during retrieval of similar pair-associates over discrimination of the actual target.

## General discussion

In the present study we compared VAM between synesthetes and non-synesthetes in two different age groups, using a novel between-group design. Synesthetes were found to have an associative learning and retrieval advantage, even for stimuli that do not elicit a synesthetic color experience. Specifically, our findings yielded a significant difference between synesthetes and older controls, but no differences between synesthetes and younger adults or between younger and older adults. This suggests that there is a small difference between synesthetes and younger adults that most experiments would be unable to detect without a highly impractical increase in subject numbers. This small, albeit non-significant, advantage of synesthetes over young controls was evident in the learning rate (Figure 3), and memory performance for both similar and dissimilar pairs (Figures 4, 6).

The results shed light on previous inconsistent findings of a memory advantage in synesthetes for achromatic abstract stimuli (Rothen and Meier, 2010; Gross et al., 2011), given that the memory advantage of young synesthetes is too subtle to be reliably detected relative to age-matched controls, but emerges in comparison to older adults. Rothen et al. (2012) recently offered an explanation for the synesthetes' memory advantage on the basis of the representational memory account. According to this account, visual stimuli are processed by the same neural substrates along the ventral visual stream as they are being retrieved from memory, suggesting a perceptual-mnemonic continuum of visual stimulus processing (Bussey and Saksida, 2007; Saksida and Bussey, 2010). The characteristics of grapheme-color synesthesia satisfy particularly well the stimulus-dependent processing operations suggested by the representational memory account. First, the synesthetes' subjectively experienced colors in response to verbal stimuli encompass two features (colors, letters) that are both represented in the ventral visual stream. Second, the perceptual letter-to-color associations lead to improved memory for verbal stimuli in synesthetes (Yaro and Ward, 2007; Rothen and Meier, 2010; Radvansky et al., 2011), thus supporting the representational memory account of a perceptual-mnemonic continuum. Specifically, the verbal memory advantage supports the dual-coding theory, suggesting that when letters trigger colors, stronger memory representations are elicited in the same neural substrate. The representational account further supports the color-expertise hypothesis (Pritchard et al., 2013): if there is a perceptual-mnemonic continuum, the synesthetes enhanced color perception (Banissy et al., 2009) should feed into enhanced color memory. Thus, when color is a constituent feature in abstract shapes, it is this feature for which synesthetes show greatest associative memory, over shape or location (Pritchard et al., 2013).

Here, we have shown an associative memory advantage in synesthetes over older adults for achromatic abstract stimuli, suggesting additional differences in the synesthetic brain which facilitate memory functions. Indeed, the evidence suggests differences in the synesthetes' anatomical and functional circuitry relative to controls that are often found along the ventral visual stream (see Rouw et al., 2011 for review). Processing of achromatic abstract shapes can be traced to even more posterior visual regions in the brain, as early as primary visual cortex. Given that synesthetes were found to show perceptual processing differences for achromatic abstract stimuli in early visual cortex (Barnett et al., 2008; Terhune et al., 2011), it is plausible, according to the representational memory account, that such early perceptual processing differences equally potentiate memory for these stimuli. This could explain the differences between synesthetes and young adults found in the present study, which were too subtle to yield a significant memory advantage.

How can we explain the synesthetes memory advantage over older adults? One explanation is the altered white-matter microstructure in synesthetes that has been observed in parietal, frontal and temporal areas of the brain (Rouw and Scholte, 2007; Whitaker et al., 2014), suggesting altered connectivity across the synesthetic brain (see also Hanggi et al., 2011). By contrast, the brain of older adults is frequently characterized by white matter injury (Lockhart et al., 2012), or white matter atrophy (Vernooij et al., 2008), suggesting that the structural integrity, and thus, connectivity breaks down in old age. These anatomical differences are related to cognitive function and have shown, for instance, an age-related association between white matter integrity and enhanced perceptual discrimination of faces (Thomas et al., 2008), as well as an association between white matter injury in older adults and poorer VAM (Lockhart et al., 2012). By contrast, a recent study by Whitaker et al. (2014) has shown a correlation between synesthetes' white matter structure and their self-reported vividness of visual imagery, such that synesthetes with more crossing fibers experienced greater visual imagery. These findings suggest that the pervasive structural brain differences in synesthetes and older adults may have brought about the behavioral associative memory differences, which were too subtle to detect against young adults.

With respect to aging, an interesting observation was the non-significant difference between young and older adults in the *d* prime scores of sensitivity, especially in the dissimilar retrieval condition that requires high levels of discriminability. Previous associative recognition tests have shown a significant memory reduction in older relative to young adults, characterized by older adults' frequent false alarm responses (Naveh-Benjamin et al., 2004, 2009; Cohn et al., 2008; Shing et al., 2008; Edmonds et al., 2012). Specifically, these false alarm responses were attributed to age-related difficulties in discriminating match trials from non-match trials due to increased reliance on picture familiarity. In the present study, we have shown that this issue can be alleviated when the initial learning phase is self-paced, allowing sufficient time to encode the pair-associates. In practical terms, this suggests that age-related memory problems might be reduced by investing more time in associative learning.

Two limitations of the present study should be mentioned. First, the relatively small sample size of 14 participants in each group has to some degree affected the generalizability of the data, as shown by our reported power calculations. Importantly however, the underpowered results were mostly found between young and older adults, suggesting that with increased sample sizes we would have been able to demonstrate a significant memory advantage in young vs. older adults, a finding that is not new. The more critical results however pertained to the differences found between synesthetes and older adults, all of which demonstrated sufficient power in the dissimilar memory conditions. Second, it could be argued that our learning and retrieval paradigm might not be sensitive enough to detect the differential effects of aging and synesthesia (e.g., in the similar conditions). Ongoing work in our lab currently involves a four-alternative-forced-choice trial-and-error learning paradigm that might increase the sensitivity in detecting age and individual differences on the number of Runs required during pair-associative learning, as well as the effectiveness of this paradigm on subsequent retrieval. A final limitation that cannot be ruled out, and is shared by the majority of studies with synasthetes, is the issue of motivational differences; namely the fact that the synasthetes know they have been invited on the study because of their synesthesia.

In conclusion, this study shows that associative memory advantages are observed in synesthetes even with achromatic abstract, non-synesthesia-inducing stimuli. However, the advantages are subtle and can only be detected in comparison to older adults. Crucially, our results indicate that perceptual mechanisms (enhanced in synesthesia, declining with aging) may contribute to a generic associative memory advantage, and may help explain the deficits in associative memory that occur with healthy aging.

### Conflict of interest statement

The authors declare that the research was conducted in the absence of any commercial or financial relationships that could be construed as a potential conflict of interest.
